# Complaints as starting point for vicious cycles in customer–employee-interactions

**DOI:** 10.3389/fpsyg.2015.01454

**Published:** 2015-10-16

**Authors:** Eva Traut-Mattausch, Sara Wagner, Olga Pollatos, Eva Jonas

**Affiliations:** ^1^Department of Psychology, University of Salzburg, SalzburgAustria; ^2^Department of Psychology, University of Munich, MunichGermany; ^3^Department of Psychology, University of Ulm, UlmGermany

**Keywords:** self-esteem threat, defense response, customer complaint, customer–employee-interaction, motivated cognition, motivated behavior

## Abstract

A ring-model of vicious cycles in customer–employee-interaction is proposed: service employees perceive complaints as a threat to their self-esteem resulting in defense responses such as an increased need for cognitive closure, a devaluation of the customer and their information and degrading service behavior. Confronted with such degrading service behavior, customers react defensively as well, by devaluing the employee for example with regard to his/her competence and by reducing repurchase and positive word-of-mouth (WOM). Three studies investigated each link in this ring-model. In study 1, participants were confronted with an aggressive or neutral customer complaint. Results show that motivated closed-mindedness (one aspect of the need for cognitive closure) increases after an aggressive complaint leading to a devaluation of the customer and their information, and in turn to a degrading service reaction. In study 2, participants were confronted with a degrading or favorable service reaction. Results show that they devaluate the employees’ competence after receiving a degrading service reaction and thus reduce their intention to repurchase. In study 3, we finally examined our predictions investigating real customer–employee-interactions: we analyzed data from an evaluation study in which mystery callers tested the service hotline of an airline. Results show that the employees’ competence is devaluated after degrading behavior and thus reduces positive WOM.

## Introduction

Several weeks ago, Betty went to the local office of a travel agency and booked holiday package for Paris at a favorable price. As a customer she was very satisfied with the service and therefore recommended the travel agency to friends and colleagues. Due to an urgent project at work, however, she now has to change her travel arrangements and leave a few days later than planned. Therefore, she called the hotline of the travel agency and had her tickets changed without any problems. Yesterday, however, she received an invoice charging an extra fee for changing the flights—something she had not been told by the employee of the hotline. In fact, she had even asked for a flexible ticket in the beginning in order to avoid extra charges in case of changes. Betty is surprised about the invoice and calls the hotline of the travel agency a second time. Having waited several minutes, she confronts the service employee, John, with her problem and complains about the extra charge. Furthermore, she says: “I do not want to pay it.” John feels stressed because of the complaint. In fact, he is not willing to dispute about the problem based on non-reliable information from an uninformed and incompetent customer like Betty and therefore replies: “Now don’t you pretend you did not know that you could not change budget flights for free. Only because you do not want to pay for this, you cannot blame us for your mistake.” Confronted with this degrading service reaction, Betty devaluates John’s professional competence, decides to never book again with this travel agency and not to recommend their service anymore. She tells John: “I’m not responsible for this!” and complains again about her problem, the nasty business practices, and his degrading service reaction as well…

Research shows that this customer–employee-interaction might be quite realistic. For service employees, like John, the customers’ behavior is quite challenging from time to time, especially in case of complaints (Richins, 1983). On the other side customers, like Betty, often remain dissatisfied after they have complained due to inadequate responsiveness (Naylor, 2003). For the hotel sector, Lewis and McCann (2004) report that almost 50% of all customers who had complained are dissatisfied with the service recovery. In their research about complaint management, Holloway and Beatty (2003) found that 58% of all complainants remain dissatisfied with the recovery, a majority of which blame it on poor customer service and poor interaction during the complaint encounter. So what exactly happens during the interaction when a service employee, like John, is confronted with a complaint and leaves the customer, like Betty, dissatisfied with the complaint management?

**FIGURE 1:**
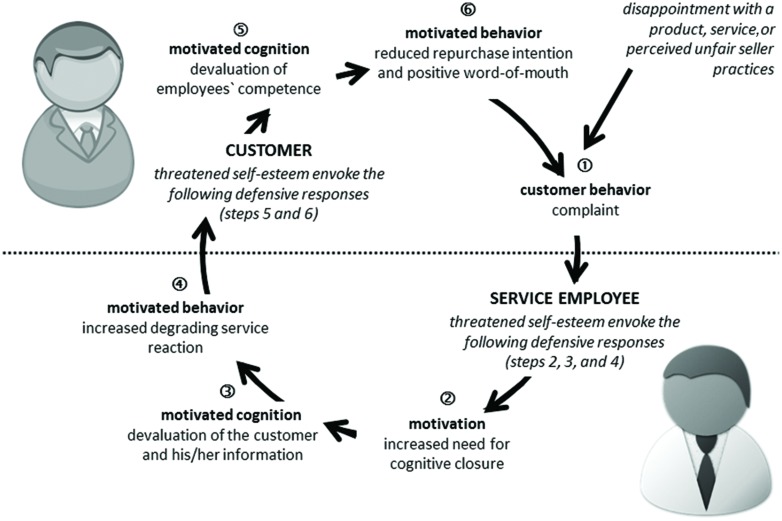
**A ring-model of vicious cycles of customer–employee-interaction**.

To answer this question it is important to focus on the *customer–employee-interaction*. Like all dyadic interactions, service encounters are a dynamic interactive process between customer and service employee (Rafaeli et al., 2008). Both interaction partners influence one another—the customer influences the service employee whose reaction in turn influences the customer and so forth. Based on the Loop2Loop model of social interactions (Jonas, 2015; Jonas and Steindl, 2015; Jonas and Bierhoff, in press), we investigated a ring-model of vicious cycles in customer–employee-interaction within the current research. The Loop2Loop model subdivides the interaction process between two persons into single steps. During the interaction between Person A and Person B, A’s behavior affects B’s emerging motivational-affective state which then leads to B’s motivated cognition and motivated behavior toward A. Accordingly, B’s behavior affects A’s emerging motivational-affective state and thus the following motivated cognition and motivated behavior toward B and so forth. The relationship-loop can develop in a positive way. However, it can also result in a negative dynamic as soon as one’s motive is threatened during the interaction process. Accordingly, a negative motivational-affective state arises, followed by negative cognitions and resulting in a negative motivated behavior which in turn possibly threatens the motive of the other interacting person. We transferred the steps described within the Loop2Loop model—motivation, motivated cognition, motivated behavior—to the ring-model of vicious cycles in customer–employee-interaction. The ring-model is illustrated in **Figure 1**.

### The ring-model of Customer Complaints

Our proposed ring-model starts with the customer behavior when the complaint is brought forward by the customer (see step 1 illustrated in **Figure 1**). According to Kowalski (1996), a complaint is a behavioral expression of dissatisfaction and could be based on a disappointment with a product, service, or perceived unfair seller practices (Fornell and Westbrook, 1979). Complaining customers provide feedback that their expectations were not met which could be valuable information to improve products or service quality (Kowalski, 1996; Traut-Mattausch and Jonas, 2007). An example for this could be Betty, our customer, who complains about the extra charge. This is a negative feedback for her interaction partner John, the service employee, because Betty’s complaint indicates that her expectations were not met during the service process. John could use this indication to improve the service process, if he reacted in an open-minded way: by asking for details in order to understand underlying reasons and by appreciating Betty’s information and suggestions. In our short story, however, John reacts quite differently, in a closed-minded way. He questions Betty’s competence, devalues her information, and shows a degrading service reaction. Why is this?

**FIGURE 2:**
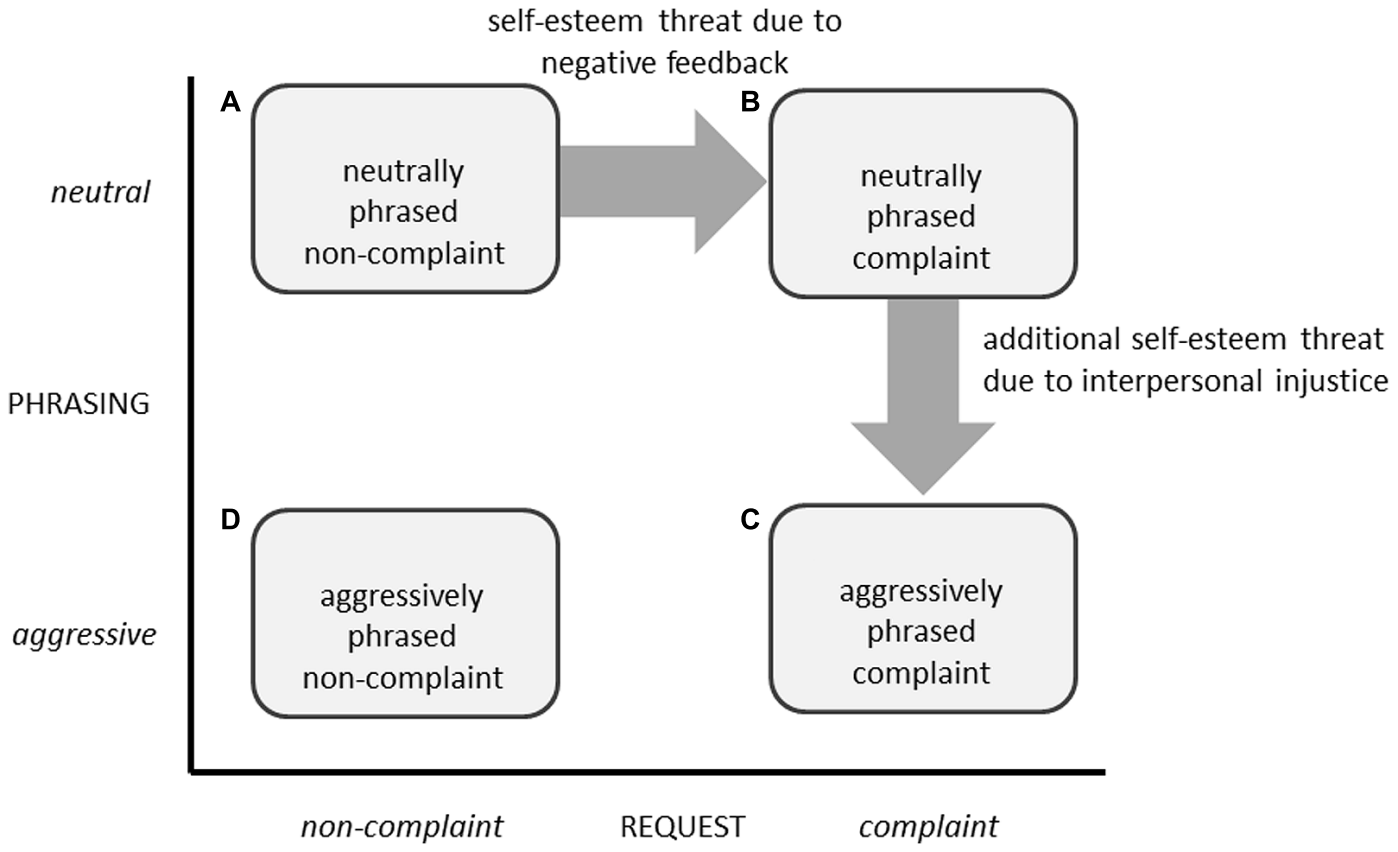
**Four types of behavioral expressions.** The combination of request (non-complaint vs. complaint) and phrasing (neutral vs. aggressive) results in four types of behavioral expressions: **(A)** neutrally phrased non-complaints (e.g., customer to a service employee of a travel agency: “Thank you for your offer. I’m a frequent flier with this airline. Is there any chance to get a price reduction due to my high amount of bonus miles?”), **(B)** neutrally phrased complaints (e.g., “The extra charge must be a mistake. I do not want to pay it for the change of my booking.”), **(C)** aggressively phrased complaints (e.g., “What nasty business practices! I won’t even consider paying this extra charge.”), and **(D)** aggressively phrased non-complaints (e.g., “The offer is an absolute scream! I’m a frequent flier with a high amount of bonus miles and therefore expect a fair and favorable price. Otherwise I want to talk to your boss!”).

In order to predict John’s reaction we have to look at the threatening effects of complaints. As already mentioned a complaint can be seen as *negative feedback* expressing a disappointment (Kowalski, 1996) and therefore different from other customer requests such as asking for product information and offers or negotiating about prices and interests (see dimension *request* illustrated in **Figure 2**). Only negative customer feedback challenges the positive view about oneself (e.g., complaining about incomplete information or a wrong recommendation) or one’s group (e.g., complaining about a faulty product, about a delayed delivery process, or bad business practices) and therefore threatens the self-esteem of a service employee which in turn leads to defense responses (Traut-Mattausch and Jonas, 2007). Empirical evidence investigating the effect of positive compared to negative feedback supports this idea. Feedback is not only uncomfortable for the receiver but even a threat toward the receiver’s own self-worth (Leung et al., 2001). Therefore, defensive reactions toward (negative) feedback can be shown: it is, for example, less accepted and perceived as less accurate than positive feedback (Snyder and Newburg, 1981; Fedor et al., 1989)—even if the feedback sender tries consciously not to offend the receiver (Argyris, 1985, 1991). Negative feedback can be seen as self-esteem threatening information about one’s own perceived inadequacy. A devaluation of the feedback and/or its source is a useful defense for one’s own self-worth (Leung et al., 2001). We therefore conclude that a complaint is a threat toward the self-esteem of an employee—even if it is neutrally phrased.

Additionally, complaints (as well as other requests, “non-complaints”) can be phrased either neutral or aggressive by the customer (see dimension *phrasing* illustrated in **Figure 2**). For example Betty’s complaint referring not only on one case of bad customer service but also to “nasty business practices in general.” Let us now imagine that the situation escalates and Betty shouts furiously: “I won’t even consider paying this extra fee! I want to speak to your boss right now!” As research shows this is a quite realistic scenario and makes customer service challenging for employees like John, especially in case of complaints. Richins (1983) distinguished four types of customer interaction styles, which occur in case of complaints—non-assertive, assertive, resort-to-aggression, and aggressive. Unfortunately, from John’s perspective, only non-assertive customers refrain from complaining, whereas all other types of customers complain rather frequently (Richins, 1983). Thus, complaint management contains a good deal of unpleasant interactions with rather aggressive customers. Grandey et al. (2004) even report that call center employees, like John, are verbally attacked by customers 10 times on an average working day. Not to be treated politely, and with dignity and respect by others (*interpersonal justice*, Greenberg, 1993) is harmful, because mistreatments—such as using abusive words and actions—are signals for disrespect (Bies, 2001). Furthermore, Ferris et al. (2012) pointed out that such interpersonal injustice conveys threatening self-relevant information and thus could cause a loss in self-esteem. Examples for such threatening self-relevant information are to be inferior, to occupy a low-status or low-value position (Lind and Tyler, 1988; Lind, 2001). Interpersonal injustice—such as accusation or insults directed against the employee—can act as a threat to self-esteem (e.g., Baumeister et al., 1996; Zdaniuk and Bobocel, 2012). Complaining customers tend to imply aggressive interaction styles (Richins, 1983) such as verbally attacking service employees (Grandey et al., 2004). Based on this interpersonal unjust treatment, we conclude that an aggressively phrased complaint should be even more threatening than a neutrally phrased complaint.

#### Negative Feedback as Threatening Aspect of Complaints: Pilot Study 1

According to practice reports employees feel most uncomfortable when dealing with complaint situations and try to avoid them (e.g., Stauss and Seidel, 2007). This is no surprise under the assumption that a complaint should threaten the self-esteem of a service employee. We argue that complaints are threatening because the service employee is confronted with negative feedback. To test this assumption we conducted a *pilot study*. Participants (*N* = 28 students at the University of Munich) were asked to put themselves into the role of an employee (for order processing, public relations, and customer service) in an office furniture department in a big department store. All participants were introduced to the situation of receiving an e-mail from the customer Mr. Herbert and informed that the customer would make suggestions in order to improve the order processing. Therefore, all participants expected a customer feedback. However, in the *negative feedback condition* participants were additionally informed that the customer complains extensively about a problem with the order processing, whereas in the *neutral feedback condition* participants were informed that the customer purchases office furniture. According to the Kano-model a customer should be extremely dissatisfied as soon as “must-be” requirements are not fulfilled. Customers take these requirements for granted and do not ask for them beforehand (Matzler and Hinterhuber, 1998). As a consequence, complaints are expressed as negative feedback by customers. This negative feedback is one source to learn more about the “must-be” requirements of a product or service performance (Seder and Al Hazza, 2014). Within the Kano-model “must-be” requirements are differentiated from “one-dimensional” requirements. Customer satisfaction is proportional to the level of fulfillment of these requirements. “One-dimensional” requirements are usually expressed by a customer beforehand (Matzler and Hinterhuber, 1998). For us this behavior would be expressed as neutral feedback and correspond to the type (A) neutrally phrased non-complaint (see **Figure 2**). In the negative feedback condition participants were informed that the customer complains extensively about a problem. Because participants did not receive the complaint they were not confronted with verbal attacks. Thus, for us the manipulation of negative feedback corresponds to the type (B) neutrally phrased complaint (see **Figure 2**). We therefore compared within the first pilot study type (A) neutrally phrased non-complaints (condition “neutral feedback”) with type (B) neutrally phrased complaints (condition “negative feedback”).

Participants were asked to indicate their actual feelings in order to measure psychological discomfort by using seven items from the psychological discomfort scale (Elliot and Devine, 1994): good (recoded), uncomfortable, friendly (recoded), anxious, bothered, optimistic (recoded), happy (recoded) (α = 0.84). All items were measured on a 7-point scale from 1 (*not at all*) to 7 (*very much*). Following Balcetis and Dunning (2007), who demonstrated that the manipulation of threat (walking across the courtyard wearing an embarrassing costume) increases psychological discomfort, we chose psychological discomfort as our indicator for threat response. Results revealed that participants felt more psychological discomfort when expecting negative customer feedback (*M* = 3.67, *SD* = 1.10) compared to a neutral customer feedback (*M* = 2.69, *SD* = 1.34), *F*(1,26) = 4.46, *p* = 0.044, η^2^ = 0.15. This result demonstrates that negative feedback could be a threatening aspect of customer complaints.

#### Aggressive Phrasing as Threatening Aspect of Complaints: Pilot Study 2

An aggressive phrasing should be another threatening aspect of customer complaints. Therefore, we conducted a *second pilot study* to test this proposition and investigated the effect of neutrally vs. aggressively phrased complaints on physical stress measured by heart rate (HR) and skin conductance level (SCL)^1^. Therefore, we compare within the second pilot study type (C) aggressively phrased complaints with type (B) neutrally phrased complaints (see **Figure 2**). We furthermore chose HR and SCL as additional indicators for threat response, because Schmid and Schmid Mast (2013) demonstrated that the manipulation of threat (social evaluation situation) increases HR and Wood et al. (2014) demonstrated that the manipulation of threat (loud white-noise) increases SCL. We hypothesized that the mean HR as well as SCL should increase to a higher extent after an aggressively phrased complaint compared to a neutrally phrased complaint. Similar to the first pilot study, participants (*N* = 14 students at the University of Munich) were asked to put themselves into the role of an employee in an office furniture department. All participants were confronted with an aggressively phrased complaint as well as with a neutrally phrased complaint after a baseline measure of HR and SCL. In support of our assumption, results revealed that HR increased significantly more after an aggressively phrased complaint (*M* = 76.65 beats per minutes, *SD* = 9.13) compared to a neutrally phrased complaint (*M* = 74.31 beats per minutes, *SD* = 9.88), *F*(1,13) = 5.26, *p* = 0.039, η^2^ = 0.29, and that SCL tended to increase more after an aggressively phrased complaint (*M* = 3.08 μS, *SD* = 1.14) compared to a neutrally phrased complaint (*M* = 2.69 μS, *SD* = 1.06), *F*(1,13) = 4.48, *p* = 0.054, η^2^ = 0.26. These results indicate that an aggressive phrasing could be another threatening aspect of customer complaints.

#### Employees’ Defense Responses

The theory of lay person epistemology predicts a high need for cognitive closure when the situation is difficult, exhausting, or uncomfortable (Kruglanski, 1989, 2004). This need is defined as an “individual’s desire for a firm answer to a question” (Kruglanski, 2004, p. 6). A high need for cognitive closure evokes a closed-minded-attitude toward new information (Kruglanski, 2004). Consequently, social judgments were made on readily accessible person schemas (Pierro and Kruglanski, 2008). An employee’s schema of a complaining customer could be “a short-tempered nagger who wants something for free” leading to biased judgment. Recent research demonstrated that the manipulation of threat increases aspects of the need for cognitive closure leading to defense responses. More precisely, Thórisdóttir and Jost (2011) showed that the manipulation of threat (Studies 1a and 1b: recall of a high amount of threatening life experience) motivated closed-mindedness (one aspect of the need for cognitive closure). Moreover, Agroskin and Jonas (2013) demonstrated that the manipulation of threat (Study 3: mortality salience and control deprivation) increases the need for structure (another aspect of the need for cognitive closure) followed by ingroup defense for people with low self-esteem. We therefore predict within our ring-model an increased *need for cognitive closure* as *motivation* after a self-esteem threatening customer complaint (see step 2 illustrated in **Figure 1**). However, as need for cognitive closure and avoidance motivation are related (Agroskin, unpublished doctoral dissertation) avoidance motivation could play a further role during the customer–employee-interaction. Avoidance motivation is defined “as the energization of behavior by, or the direction of behavior away from, negative stimuli (objects, events, possibilities)” (Elliot, 2006, p. 112). Both pilot studies demonstrated that a complaint situation could be seen as a negative stimulus resulting in an increased psychological discomfort or psychosocial stress response. Therefore, it could be assumed that complaints can elicit an avoidance motivation as an additional motivation. However, in our research we focused on the need for cognitive closure as motivation within the ring-model (see **Figure 1**).

Given that employees are motivated to maintain a positive view of themselves (Tesser, 2000), they should strive to restore their threatened self-esteem (Zdaniuk and Bobocel, 2012). For example by using biased judgments, such as the devaluation of the customer and his/her information. Consistent with this idea, several social psychological theories (e.g., motivated reasoning approach, Kunda, 1990; multiple motive approach of the heuristic systematic model, Chaiken et al., 1989; biased hypothesis testing model, Pyszczynski and Greenberg, 1987) have illustrated that people defend their self-esteem relevant positions and perceptions against attacks and criticism by processing information selectively. This process makes it possible to protect one’s self-esteem and to sustain one’s perspective of the world. Therefore, a self-esteem-beneficial hypothesis will be confirmed (e.g., the products of the own company are of high quality and do not need improvements) and the self-esteem-threatening hypothesis will be avoided (e.g., complaint and complaining behavior are legitimate). Accordingly, the customer as well as the customer’s feedback should be evaluated in a biased way. Negative feedback should be devalued in order to defend the employee’s threatened self-esteem. Therefore, our ring-model predicts that the employee’s increased need for cognitive closure, as a desire for a firm solution with regard to the complaint situation, should be followed by a *devaluation of the customer and his/her information* as a result of the employee’s *motivated cognition* (see step 3 illustrated in **Figure 1**).

For Davidow (2000, 2003) a favorable complaint handling by employees includes embracing attentiveness (i.e., listening carefully to the complainant) and credibility (i.e., explaining the problem). Based on our assumption that employees should devaluate the complaining customer and his/her information in order to boost their threatened self-esteem, we thus predict that the employee reacts in an unfavorable way, i.e., with degrading service behavior in a closed-minded manner (e.g., not listening to the complaining customer, not-accepting any inconvenience, making the customer responsible for the problem). This degrading service reaction could be a form of self-affirmation, a common response to self-esteem threats (Steele, 1988), because it symbolizes the employee’s dominance and therefore affirms the employee’s self-esteem to the extent of being superior to the victim (Baumeister et al., 1996)—in case of complaints being superior to the complaining customer. To sum it all up, we predict within our ring-model that the devaluation of the customer and his/her information should be followed by *increased degrading service reaction* toward the customer as a result of the employee’s *motivated behavior* (see step 4 illustrated in **Figure 1**).

#### Customers’ Defense Responses

As already mentioned we consider the customer–employee-interaction as a dynamic process of interaction. Complaining customers expect to be treated politely and with dignity and respect by employees (interpersonal justice, Greenberg, 1993). A favorable complaint handling (Davidow, 2000, 2003) would meet these expectations. A degrading service reaction, however, should challenge the customer’s interpersonal justice motive. Furthermore, the complaining customer receives threatening self-relevant feedback (Lind and Tyler, 1988; Lind, 2001), for instance “you are not worth being listened to carefully, we do not accept your inconvenience and you are the problem not our service,” and might perceive a loss in self-esteem (Ferris et al., 2012). We therefore conclude that degrading service behavior poses a threat toward the customer’s self-esteem and leads to defense responses by the customer. Within our proposed ring-model of vicious cycles in customer–employee-interaction (see **Figure 1**) we differentiate between a customer’s cognitive and a behavioral response.

With regard to the *motivated cognition* we predict an increased *devaluation of employees’ competence* after degrading service behavior (see step 5 illustrated in **Figure 1**). This prediction could be derived from the above mentioned motivation to maintain a positive view of oneself (Tesser, 2000) resulting in an attempt to restore the threatened self-esteem (Zdaniuk and Bobocel, 2012), for example by devaluating the employee’s competence. This prediction is in line with “motivated cognition”-approaches (e.g., Pyszczynski and Greenberg, 1987; Kruglanski, 1989; Kunda, 1990) pointing out that people defend important self-esteem relevant perspectives against attacks and critiques, and distort information in respect of this defense, for example by devaluating the criticism itself or its source.

The last step of our ring-model predicts the devaluation of the employee’s competence followed by *reduced positive word-of-mouth* (WOM) and a *reduced repurchase intention* as a result of the customer’s *motivated behavior* (see step 6 illustrated in **Figure 1**). Positive WOM is defined as the likelihood of customers spreading favorable information about an organization, which includes recommending the organization and its products and services (Maxham and Netemeyer, 2003) whereas repurchase intention is defined as the intention to continue to do business with an organization (Blodgett et al., 1997). Positive WOM usually occurs after positive and satisfying experiences (Parasuraman et al., 1988; for an overview see de Matos and Rossi, 2008), when customers’ expectations were met or even exceeded (Maxham and Netemeyer, 2002). These expectations are competent and helpful service behavior and the feeling that employees take them seriously (Zeithaml and Bitner, 2000; Gruber et al., 2006). In this line competence could be identified as one key driver for positive WOM in laboratory experiments (Johnson and Zinkhan, 1991; Johnson et al., 1998). For the financial service sector, Rajaobelina and Bergeron (2009) report expertise and customer orientation as antecedents for a good relation between employee and customer which in turn positively influence repurchase intentions and positive WOM. Furthermore, negative interpersonal experiences reduce the intention to spread positive WOM (for an overview see Richins, 1983; Gremler and Gwinner, 2000; Gremler et al., 2001). Therefore, we predict a decreased positive WOM and decreased repurchase intention triggered through the devaluation of employees’ competence during the service interaction within our ring-model.

### Overview of Studies

We conducted two laboratory studies and a field study to test each link of our proposed ring-model of vicious cycles in customer–employee-interaction. At first, study 1 investigated the motivation (step 2), the motivated cognition (step 3), and the motivated behavior (step 4) provoked by an aggressively phrased complaint compared to a neutrally phrased complaint (step 1). Study 2 then investigated the motivated cognition (step 5) and the motivated behavior (step 6) provoked by degrading compared to favorable service behavior (step 4). Furthermore, we conducted a field study investigating real customer–employee-interactions (study 3) to show evidence for our proposed ring-model. Whereas we compared (C) aggressively phrased complaints to (B) neutrally phrased complaints in study 1, we compared (C) aggressively phrased complaints to (A) neutrally phrased non-complaints in study 3 (see **Figure 2**)^2^.

## Study 1

The aim of study 1 was to test the hypothesized steps 1–4 assumed within the described ring-model (see **Figure 1**). More precisely, we wanted to show that participants respond with an increased need for cognitive closure (motivation), with an increased devaluation of the customer and his/her information (motivated cognition), and with an increased degrading service reaction (motivated behavior) when confronted with an aggressively phrased complaint compared to a neutrally phrased complaint. Furthermore, we hypothesized that the effect on increased degrading service behavior intention should be mediated by the need for cognitive closure (mediator 1) and the devaluation of the customer and his/her information (mediator 2).

### Method

#### Participants and Design

Fifty-eight students (42 female and 16 male) with an average age of 23.33 years (*SD* = 3.28) of the University of Munich participated in this study. The design was a 2 (*complaint phrasing*: neutral vs. aggressive) × 2 (*service reaction*: favorable vs. degrading) factorial design with repeated measure on the second factor. Participants were randomly assigned to the experimental conditions aggressive vs. neutral phrasing. We balanced the order in which both service reactions—favorable and degrading—were presented^3^.

#### Procedure and Material

Participants were asked to put themselves in the place of service employees of an airline. In this role, they read the statement of a customer, who called their service hotline and refused paying an extra charge for the change of a booking. For one half of the participants the customer complaint was phrased aggressively; for the other half the customer complaint was phrased in a neutral way.

(*neutrally* phrased complaint) I do not want to pay an extra charge for the change of my booking. I called beforehand and asked whether it is possible to fly 2 days later. This would have been the perfect time to tell me that I cannot change my bookings for free. All in all, the booking process was too fast, so I did not have time to read through your terms of business. But this is exactly why I called your hotline: to get detailed information. It is not fair to inform me about this extra charge now.

(*aggressively* phrased complaint) I won’t even consider paying an extra charge for the change of my booking! I called beforehand and asked whether it is possible to fly 2 days later. You should have told me that I cannot change my bookings for free. But you were so keen to come to an end that I did not have enough time to read through your terms of business. If I take extra time to call your hotline I expect excellent advice, but you are obviously not capable. To inform me about this extra charge now is monkey business!

Then, participants were asked the following two items as a manipulation check: (a) how much do you feel the customer offends you and (b) how polite is the customer (recoded). Next, participants were asked a single item in order to measure the aspect “closed-mindedness” of the need for cognitive closure as mediator one—how much would you like to talk to the customer (recoded)^4^—and the following eight items in order to measure the devaluation of the customer and his/her information as mediator two: (a) how competent is the customer (recoded), (b) how reliable is the customer (recoded), (c) how intelligent is the customer (recoded), (d) how appropriate is the customer’s behavior (recoded), (e) how credible is the customer (recoded), (f) how informative is the customer’s call (recoded), (g) how seriously do you take the information (recoded), and (h) how reliable is the information (recoded).

Furthermore, participants were presented a favorable and a degrading service reaction.

(*favorable* service reaction) Unfortunately, this was not going very well. You are right, we should pay more attention to explain our terms of business before our customers make their bookings. It has to be transparent for our customers and give them enough time to reconsider their decision.

(*degrading* service reaction) Now do not you pretend you did not know that you could not change budget flights for free. Only because you do not want to pay for this, you cannot blame us for your mistake.

After each service reaction participants were asked how much they would like to react in this way. All items were measured on a 6-point scale from 1 (*not at all*) to 6 (*very much*).

### Results

#### Manipulation Check

The intercorrelation of the two manipulation check items was high enough to allow both items to be compiled to an overall measure (*r* = 0.51, *p* < 0.001). The mean for this measure was significantly higher in the aggressive phrasing condition (*M* = 4.54, *SD* = 0.94) compared to the neutral phrasing condition (*M* = 3.10, *SD* = 0.98), *F*(1,56) = 32.36, *p* < 0.001, η^2^ = 0.37. This result indicates that the manipulation of the complaint phrasing was successful.

#### Closed-mindedness

To test whether closed-mindedness was higher after an aggressively phrased complaint compared to a neutrally phrased complaint we ran an ANOVA. Results revealed more closed mindedness after an aggressively phrased complaint (*M* = 4.86, *SD* = 1.24) compared to a neutrally phrased complaint (*M* = 3.83, *SD* = 1.51), *F*(1,56) = 7.90, *p* = 0.007, η^2^ = 0.12.

#### Devaluation of the Customer and his/her Information

We first created a measure by aggregating the eight items (α = 0.87). We then analyzed the influence of complaint phrasing on the devaluation of the customer and his/her information. Results revealed that the devaluation was higher after an aggressively phrased complaint (*M* = 3.86, *SD* = 0.69) compared to a neutrally phrased complaint (*M* = 3.30, *SD* = 1.00), *F*(1,56) = 6.12, *p* = 0.016, η^2^ = 0.10.

#### Service Reaction

Next, we ran a 2 (*complaint phrasing*: neutral vs. aggressive) × 2 (*service reaction*: favorable vs. degrading) ANOVA with repeated measures on the last factor. The ANOVA showed a significant main effect for the “service reaction” factor, *F*(1,55) = 16.26, *p* < 0.001, η^2^ = 0.23 (favorable: *M* = 4.02, *SD* = 1.42; degrading: *M* = 2.67, *SD* = 1.71). However, and even more importantly, we found an interaction between “complaint phrasing” and “service reaction,” *F*(1,55) = 5.84, *p* = 0.019, η^2^ = 0.10. Simple effects analyses showed that confronted with an aggressively phrased complaint participants preferred to show degrading service reaction (*M* = 3.36, *SD* = 1.73) compared to a neutrally phrased complaint (*M* = 1.97, *SD* = 1.43), *p* = 0.001. However, regarding the preference of the favorable service reaction, simple effects analyses showed no difference between an aggressively phrased complaint (*M* = 3.89, *SD* = 1.31) and a neutrally phrased complaint (*M* = 4.14, *SD* = 1.53), *p* = 0.520. This result indicates that after an aggressively phrased complaint participants preferred to show a degrading service reaction to a greater extent compared to a neutrally phrased complaint, whereas the favorable reaction did not differ depending on the complaint phrasing. Looking at it from the other way, simple effects analyses showed, that after a neutrally phrased complaint, participants preferred to show a favorable service reaction over a degrading service reaction, *p* < 0.001, whereas after an aggressively phrased complaint both service reactions were preferred equally, *p* = 0.275.

In addition, we created a difference measure by subtracting the preference for the degrading service reaction from the preference for the favorable service reaction as dependent variable.

Difference measure service reaction = preference to show favorable service reaction − preference to show degrading service reaction.

Then, we investigated the effect of complaint phrasing on the difference measure service reaction. Results revealed a greater difference after the neutrally phrased complaint (*M* = +2.14, *SD* = 2.46) compared to the aggressively phrased complaint (*M* = +0.54, *SD* = 2.54), *F*(1,55) = 5.84, *p* = 0.019, η^2^ = 0.10.

#### Mediation Analysis

**FIGURE 3:**
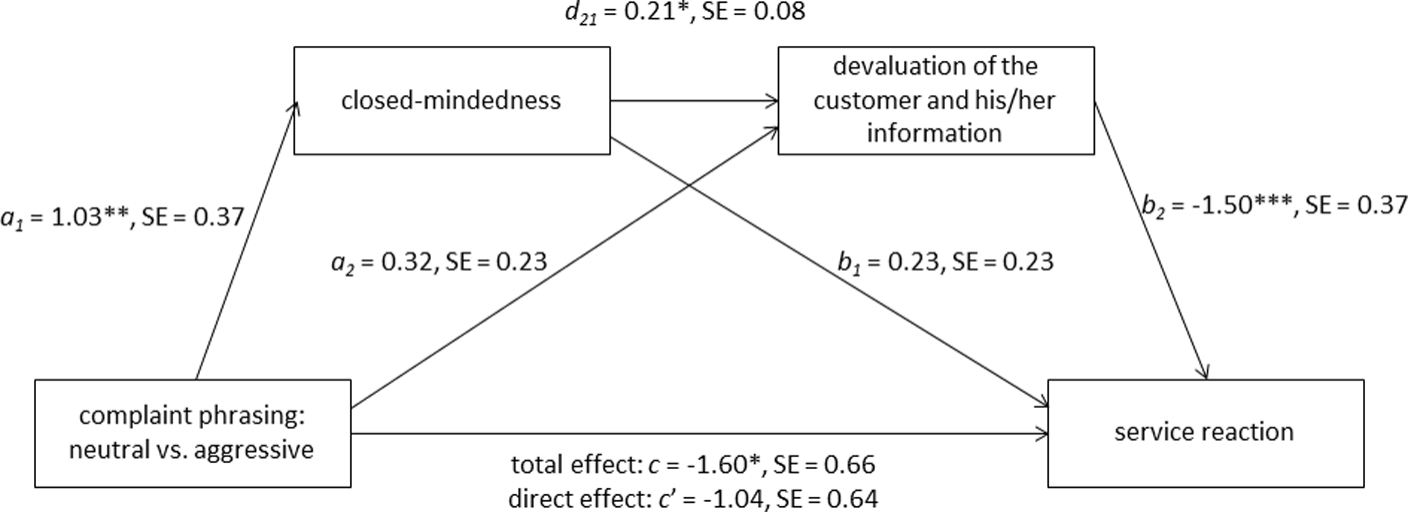
**Closed-mindedness (mediator one) and devaluation of the customer and his/her information (mediator two) mediated the effect of complaint phrasing (neutral vs. aggressive) on service reaction in study 1.** The mediation analysis was conducted using PROCESS (model 6, Hayes, 2013, p. 446). Coding of the independent variable “complaint phrasing” was neutral = 0 and aggressive = 1. The dependent variable “service reaction” is a difference measure by subtracting the preference for degrading service reaction from the preference for the favorable service reaction. *a_1_*, *a_2_*, *b_1_*, *b_2_*, *c*, *c′*, and *d_21_* are unstandardized regression coefficients. *p < 0.05, **p < 0.01, ***p < 0.001.

Moreover, we analyzed whether the effect of complaint phrasing (independent variable) on the difference measure service reaction (dependent variable) could be explained through closed-mindedness (mediator one) and devaluation of the customer and his/her information (mediator two) using PROCESS (model 6, Hayes, 2013, p. 446). The results are displayed in **Figure 3**.

The analyses revealed a significant influence of complaint phrasing on the difference measure service reaction, *B* = -1.60, *SE* = 0.66, *p* = 0.019, and the mediator one, *B* = 1.03, *SE* = 0.37, *p* = 0.007. Subsequent analyses of the mediator one on the mediator two, *B* = 0.21, *SE* = 0.08, *p* = 0.012, as well as of the mediator two on the difference measure service reaction, *B* = -1.50, *SE* = 0.37, *p* = 0.001, showed significant regression weights indicating considerable influence of mediator one on mediator two as well as of mediator two on the difference measure service reaction. When finally examining the influence of the complaint phrasing of mediator one and mediator two on the difference measure service reaction concurrently, the effect of the complaint phrasing was considerably reduced, *B* = -1.04, SE = 0.64, *p* = 0.109. The indirect effect of the complaint phrasing on the difference measure service reaction through mediator one and two was highly significant as indicated by the 95% CI (-0.92, -0.04) using 1,000 bootstrap estimations.

### Discussion

Results of study 1 support all hypothesized steps 1–4 assumed within our ring-model. An aggressively phrased complaint increased the closed-mindedness motivation compared to a neutrally phrased complaint (motivation). Consequently, the customer and the customer’s information were devalued in case of an aggressively phrased complaint compared to a neutrally phrased complaint (motivated cognition). Concerning the preferred service reactions (motivated behavior), our expectations were also confirmed. A neutrally phrased complaint resulted in increased preferences for the favorable service reaction toward the customer and reduced preferences for the degrading service reaction. This difference, however, disappeared in case of an aggressively phrased complaint, when the preference for the degrading service reaction increased to an equal level with the favorable service reaction. Finally, the mediation analysis provides evidence for the assumed role of the need for cognitive closure and the devaluation of the customer and his/her information as mediators in predicting the service reaction.

However, study 1 is limited by the fact that closed-mindedness was the only aspect of the need for cognitive closure that was measured. Therefore, we conducted a further study measuring another aspect of the need for cognitive closure—the personal fear of invalidity (Thompson et al., 2001). Fear of invalidity (avoidance of early closure) can motivate people to be accurate by a desire to avoid the dullness associated with relying on whatever thoughts come to mind first (Moskowitz, 2005). In contrast, people with *no* fear of invalidity (achievement of early closure) should be motivated to evaluate information on readily accessible person schemas. An employee’s schema of an aggressively complaining customer could be “a short-tempered nagger who wants something for free” leading to a negative evaluation of the customer and his/her information. To test the effect of fear of invalidity on the devaluation of customers’ information we asked participants (*N* = 75 students of the University of Munich) to put themselves into the role of an employee in the student counseling service of the University of Munich^5^. All participants were informed that students can evaluate the service of the counseling department through an online form. Next, all participants were asked to read several web evaluations. Each evaluation was a complaint phrased either neutrally (two evaluations) or aggressively (two evaluations) and included suggestions for the improvements of the service. In order to measure the devaluation of the suggestions we asked all participants to evaluate the suggestions by using the following three items: (a) the student provides suggestions in order to optimize the service of the counseling department (recoded), (b) the suggestions of the student are helpful (recoded), and (c) the suggestions of the student are important (recoded) (α = from 0.73 to 0.84). All items were measured on a 5-point scale from 1 (*not at all applicable*) to 5 (*very much applicable*). In the absence of a well-established *state* measure we decided to use the *trait* measure “personal fear of invalidity” (German short version, Hänze, 2002). This trait measure consists of six items such as “I tend to continue to evaluate recently made decisions” (α = 0.86). We recoded the items to measure *no* fear of invalidity which represents an achievement of early closure. Within our ring-model we assume an effect of complaints on the devaluation of the customers’ information mediated by the need for cognitive closure as a state variable. However, we measured (no) fear of invalidity as a trait. Therefore, we predicted that the effect of aggressively vs. neutrally phrased complaints on the devaluation of customers’ suggestions is moderated by the trait (no) fear of invalidity. To test this assumption we z-standardized the moderator (no fear of invalidity) and ran a repeated measure ANOVA with the within-factor devaluation (aggressively vs. neutrally) and no fear of invalidity as covariate. The ANOVA showed a significant main effect for the within factor, *F*(1,73) = 71.15, *p* < 0.001, η^2^ = 0.49 (devaluation of the aggressively phrased suggestions: *M* = 2.21, *SD* = 0.79; devaluation of the neutrally phrased suggestions: *M* = 1.49, *SD* = 0.54). However, and more important, we found an interaction between “no fear of invalidity” and the within-factor, *F*(1,73) = 4.49, *p* = 0.038, η^2^ = 0.06. To illustrate the nature of this interaction we calculated the correlation between the moderator (no fear of invalidity) and the devaluation of aggressively phrased suggestions and between the moderator (no fear of invalidity) and the devaluation of neutrally phrased suggestions, respectively. Results show a significant correlation between no fear of invalidity (achievement of early closure) and aggressively phrased complaints, *r* = 0.29, *p* = 0.013; whereas the correlation between no fear of invalidity (achievement of early closure) and neutrally phrased complaints was ns, *r* = 0.08, *p* = 0.481. Taken in conjunction, results of study 1 and the recently reported study show that different aspects of the need for cognitive closure (open-mindedness, fear of invalidity) are relevant for predicting a devaluation of the customer and his/her information depending on how this customer phrases the complaint.

The results of study 1 show that after an aggressively phrased complaint both the degrading and the favorable service reaction are equally preferred. However, it is unclear which reaction a service employee would choose in reality. As already mentioned, between approximately 50 to 60% of all customers who had complained are dissatisfied with the service recovery, a majority of which blame it on poor customer service and poor interaction during the complaint encounter (Holloway and Beatty, 2003; Lewis and McCann, 2004). Based on these facts, one can speculate that one half of service employees show degrading behavior in reality whereas the other half would not and that this difference could depend on the applied organizational culture, pre-defined display rules, and control possibilities of service quality (e.g., presence of others, recoding of phone conversation). However, future research may imply an either favorable or degrading behavior response measure to answer the question which behavioral option is actually chosen by employees.

All in all, study 1 revealed evidence for the assumed steps 1–4 within our ring-model. Now we shift our focus in the interaction from the employee to the customer. Study 2 therefore examined how complaining customers react when confronted with degrading service reaction by employees.

## Study 2

The aim of study 2 was to test all hypothesized steps 4–6 assumed within the described ring-model (see **Figure 1**). More precisely, we wanted to show that participants respond with an increased devaluation of employees’ competence (motivated cognition) and with a reduced repurchase intention (motivated behavior) when confronted with a degrading service reaction compared to a favorable service reaction. Furthermore, we hypothesized that reduced repurchase intention in case of degrading service reaction should be mediated by the devaluation of employees’ competence.

### Method

#### Participants and Design

Fifty-six consumers (39 female and 17 male) with an average age of 22.22 years (*SD* = 3.59) participated in this study based on a one factorial between-subject design with two experimental conditions (*service reaction*: favorable vs. degrading). Participants were recruited at the University of Munich and randomly assigned to one of the two experimental conditions.

#### Procedure and Material

Participants were asked to put themselves in the role of customers of an airline. In this role, they called the service hotline and refused to pay an extra charge for the change of their booking. After reading their own complaint, half of the participants were confronted with a favorable service reaction, and half of the participants were confronted with a degrading service reaction (the same service reactions as depended variable used in study 1).

(*scenario*) Please imagine you booked a flight online after having consulted a travel agency. You would now like to change your booking and are being informed that you will be charged an extra fee. However, you had not been informed about this extra fee before booking. You call the service hotline of your travel agency. The service employee responds:

(*favorable* service reaction) Unfortunately, this did not go very well. You are right we should pay more attention to explaining our terms of business before our customers make their bookings. This has to be transparent for our customers and they should be given enough time to reconsider their decision.

(*degrading* service reaction) Now do not you pretend you did not know that you could not change budget flights for free. Only because you do not want to pay for this, you cannot blame us for your mistake.

All participants were then asked the following two items as a manipulation check: (a) how open-minded do you consider this reaction and (b) how repulsive do you consider this reaction. Next, participants were asked the following two items in order to measure their devaluation of employees’ competence as a mediator: (a) the agent is competent (recoded) and (b) the agent acts in a professional manner (recoded). Furthermore, participants were asked the following three items in order to measure their repurchase intention as dependent variable: (a) would you recommend the service hotline, (b) would you like to call the hotline again, and (c) would you like to book another flight with the agency. All items were measured on a 6-point scale from 1 (*not at all*) to 6 (*very much*).

### Results

#### Manipulation Check

First we checked the manipulation of the “service reaction” factor. Therefore, we ran a 2 (*service reaction*: favorable vs. degrading) × 2 (*evaluation of the service reaction as*: open-minded vs. repulsive) ANOVA with repeated measures on the last factor. Results revealed a significant interaction between “service reaction” and “evaluation,” *F*(1,54) = 62.36, *p* < 0.001, η^2^ = 0.54. Simple-effect analyses showed that participants regarded the favorable service reaction as more open-minded (*M* = 3.74, *SD* = 1.38) compared to the degrading service reaction (*M* = 1.66, *SD* = 1.01), *p* < 0.001. Furthermore, simple-effect analyses revealed that participants evaluated the degrading service reaction as more repulsive (*M* = 5.66, *SD* = 0.86) compared to the favorable service reaction (*M* = 3.56, *SD* = 1.48), *p* < 0.001. These results indicated that the manipulation of the “service reaction” factor was successful.

#### Devaluation of Employees’ Competence

We first created a measure by aggregating the two items (*r* = 0.59, *p* > 0.001). Next, we analyzed how the service reaction influenced the devaluation of employees’ competence. Results revealed that the employees’ competence was more devaluated after the degrading service reaction (*M* = 5.48, *SD* = 0.65) compared to the favorable service reaction (*M* = 3.96, *SD* = 1.32), *F*(1,54) = 30.49, *p* < 0.001, η^2^ = 0.36.

#### Repurchase Intention

We created a measure by aggregating the three items (α = 0.72) and analyzed how the service reaction influenced the repurchase intention. Results revealed that the repurchase intention was lower after the degrading service reaction (M = 1.54, SD = 0.77) then after the favorable service reaction (M = 2.31, SD = 1.04), F(1,54) = 9.96, p = 0.003, η^2^ = 0.16.

#### Mediation Analysis

**FIGURE 4:**
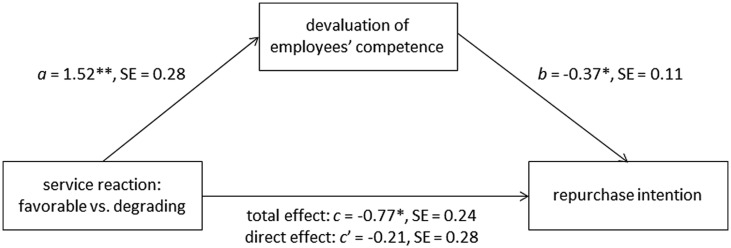
**Devaluation of employees’ competence mediated the effect of service reaction (favorable vs. degrading) on repurchase intention in study 2.** The mediation analysis was conducted using PROCESS (model 4, Hayes, 2013, p. 445). Coding of the independent variable “service reaction” was favorable = 0 and degrading = 1. *a*, *b*, *c*, and *c′* are unstandardized regression coefficients. **p < 0.01, ***p < 0.001.

Finally, we analyzed whether the effect of service reaction (independent variable) on repurchase intention (dependent variable) could be explained through the devaluation of employees’ competence (mediator) using PROCESS (model 4, Hayes, 2013, p. 445). The results are displayed in **Figure 4**. The analyses revealed a significant influence of the service reaction on the repurchase intention, *B* = -0.77, *SE* = 0.24, *p* = 0.003, and the mediator, *B* = 1.52, *SE* = 0.28, *p* > 0.001. Subsequent analyses of the influence of the mediator on the repurchase intention showed a significant regression weight, *B* = -0.37, *SE* = 0.11, *p* = 0.002, indicating considerable influence of the mediator on the repurchase intention. When finally examining the influence of the service reaction and the mediator on the repurchase intention concurrently, the effect of the service reaction was considerably reduced, *B* = -0.21, *SE* = 0.28, *p* = 0.451. The indirect effect of the service reaction on the repurchase intention through the mediator was highly significant as indicated by the 95% CI [-1.09, -0.14] using 1,000 bootstrap estimations.

### Discussion

The two hypothesized defense responses of the customer—steps 5 and 6 within the ring-model (see **Figure 1**)—were confirmed. A degrading service reaction increased the devaluation of employees’ competence (motivated cognition) and lowered the repurchase intention (motivated behavior) compared to a favorable service reaction. Furthermore, the mediation analysis provides evidence for the assumed role of the devaluation of employees’ competence as a mediator predicting the repurchase intention.

All in all, results of the first two studies revealed evidence for the assumed steps within our ring-model: Study 1 demonstrated the assumed defense responses by the employee and study 2 demonstrated the assumed defense responses by the customer. However, both studies were conducted in the lab using scenarios where participants were asked to put themselves in the role of an employee or in the role of a customer. Even though using scenarios is a usual procedure in psychological research, our studies do not demonstrate predictions derived from the proposed ring-model investigating real customer–employee-interactions. We addressed this issue in study 3.

## Study 3

For this purpose, we analyzed data from a mystery calls-evaluation study. These mystery calls were conducted in order to evaluate the service hotline of a German airline. This is a common procedure for research purposes in marketing sciences (e.g., Wilson, 1998, 2002; Finn, 2001; Van der Wiele et al., 2005). Anonymous customers, the mystery callers, pretend to be real customers in order to evaluate the service quality of a service hotline. In this evaluation study, customers—i.e., the mystery callers—brought forward a complaint or a non-complaint (neutral request) and then evaluated employees’ competence. Furthermore, they also indicated whether they would engage in positive WOM or not^6^. The nature of complaints was specified by the German airline responsible for the service quality of the service hotline (e.g., the golf equipment did not turn up during a golf holiday, changes in flight times, inconsistent cost information provided online, wrong information provided online, lost seat reservations). In case of complaints, mystery callers were asked to act aggressively and in a provocative manner toward the service employee^7^. Based on this setting we investigated whether the devaluation of service employees’ competence (as motivated cognition, step 5) is higher in case of (aggressive) complaints compared to non-complaints leading to a lowered positive WOM (as motivated behavior, step 6). We hypothesized less positive WOM in case of (aggressively phrased) complaints compared to (neutrally phrased) non-complaints. This effect should be mediated by the devaluation of employees’ competence.

More importantly, in case of complaints mystery callers should evaluate the degrading behavior of the service employee. Therefore, it is possible to investigate the hypothesized steps 4–6 within the described ring-model (see **Figure 1**) for this sub-sample. We expected to replicate the findings of study 2 and hypothesized that more degrading behavior should lead to less positive WOM and that this effect should be mediated by the devaluation of employees’ competence.

### Method

#### Sample and Design

All in all, 32 professional mystery callers conducted 160 mystery calls in order to evaluate the service hotline of a German airline. All mystery callers were blind with regard to the tested hypotheses. The design was a one factorial between-subject design with two experimental conditions (*request*: non-complaint vs. complaint). Before each call, the mystery callers randomly chose their behavior in the upcoming service encounter on request—non-complaints or complaints.

#### Procedure and Material

The content of each request (personal details, reasons for the call, etc.) were set by default in order to guarantee for standardized conditions. Mystery callers received training on the requests and the evaluation of the service employee during the call. The requests included complaints (60 calls) and non-complaints (100 calls) such as questions concerning different departments (50 calls) and flight bookings (50 calls). Two further (non-complaint) requests (specific questions concerning products and pricing in order to test selected competences of the agents) were too specific to compare it with the complaining request and therefore excluded from the data analysis. During the call, the mystery callers evaluated the competence of the service employee by answering the following three items in order to measure the devaluation of employees’ competence as mediator: (a) the agent knows the product very well (recoded), (b) the agent provides reliable information (recoded), and (c) the agent works independently and addresses problems (recoded). In case of a complaint, the following three items were used in order to measure the perceived degrading behavior of the service employee: (a) the agent recognizes the dissent (recoded), (b) the agent expresses understanding (recoded), and (c) the agent offers a solution (recoded). All items were measured on a 6-point scale from 1 (*very good*) to 6 (*very bad*). Furthermore, the mystery callers indicated whether they would recommend the service hotline or not by answering “yes” (coded 1) or “no” (coded 0) in order to measure positive WOM.

### Results

#### Devaluation of Employees’ Competence

We created a measure by aggregating the three items (α = 0.72). Next, we analyzed the influence of the request factor on the devaluation of employees’ competence. Results revealed that the employees’ competence was more devaluated after a complaint (*M* = 1.92, *SD* = 1.34) then after a non-complaint (*M* = 1.43, *SD* = 0.67), *F*(1,158) = 9.69, *p* = 0.002, η^2^ = 0.06.

#### Positive WOM

A 2 (*request:* non-complaint vs. complaint) × 2 (*recommendation:* no vs. yes) chi-square analysis was conducted. Results revealed that only 60% of the complaining customers would recommend the hotline compared to 78% of the non-complaining customers, χ^2^(1, *N* = 160) = 5.93, *p* = 0.015, eta = 0.19.

#### Mediation Analysis

**FIGURE 5:**
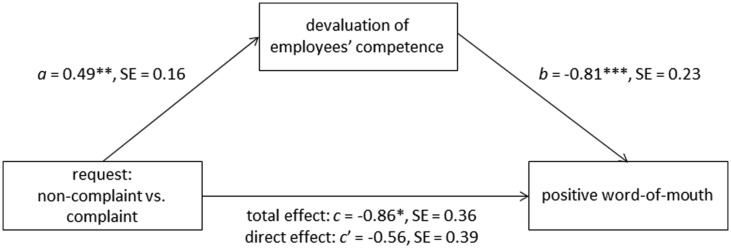
**Devaluation of employees‘ competence mediated the effect of request (non-complaint vs. complaint) on positive WOM in study 3.** The mediation analysis was conducted using PROCESS (model 4, Hayes, 2013, p. 445). Coding of the independent variable “request” was non-complaint = 0 and complaint = 1. The mystery callers indicated whether they would recommend the service hotline or not by answering “yes” (coded 1) or “no” (coded 0) in order to measure positive WOM. *a* is an unstandardized regression coefficient; *b*, *c*, and *c′* are unstandardized logistic regression coefficients. *p < 0.05, **p < 0.01, ***p < 0.001.

We then analyzed whether the effect of request (independent variable) on positive WOM (dependent variable) could be explained through the devaluation of employees’ competence (mediator) using PROCESS (model 4, Hayes, 2013, p. 445). The results are displayed in **Figure 5**. The analysis revealed a significant influence of request on positive WOM, *B* = -0.86, *SE* = 0.36, *p* = 0.016, and on the mediator, *B* = 0.49, *SE* = 0.16, *p* = 0.002. Subsequent analyses of the influence of the mediator on positive WOM showed a significant regression weight, *B* = -0.81, *SE* = 0.23, *p* < 0.001, indicating considerable influence of the mediator on positive WOM. When finally examining the influence of the request and the mediator on positive WOM concurrently, the effect of the request was considerably reduced, *B* = -0.56, *SE* = 0.39, *p* = 0.150. The indirect effect of the request on positive WOM through the mediator “employees’ competence” was highly significant as indicated by the 95% CI (-0.89, -0.11) using 1,000 bootstrap estimations.

#### Mediation Analysis (Sub-sample Complaints, *n* = 58^8^)

We created a scale by aggregating the three items measuring degrading behavior (α = 0.66). Degrading behavior was significantly related to positive WOM, *r* = -0.27, *p* = 0.043, indicating that the more degrading behavior participants perceived the less positive WOM they would spread.

**FIGURE 6:**
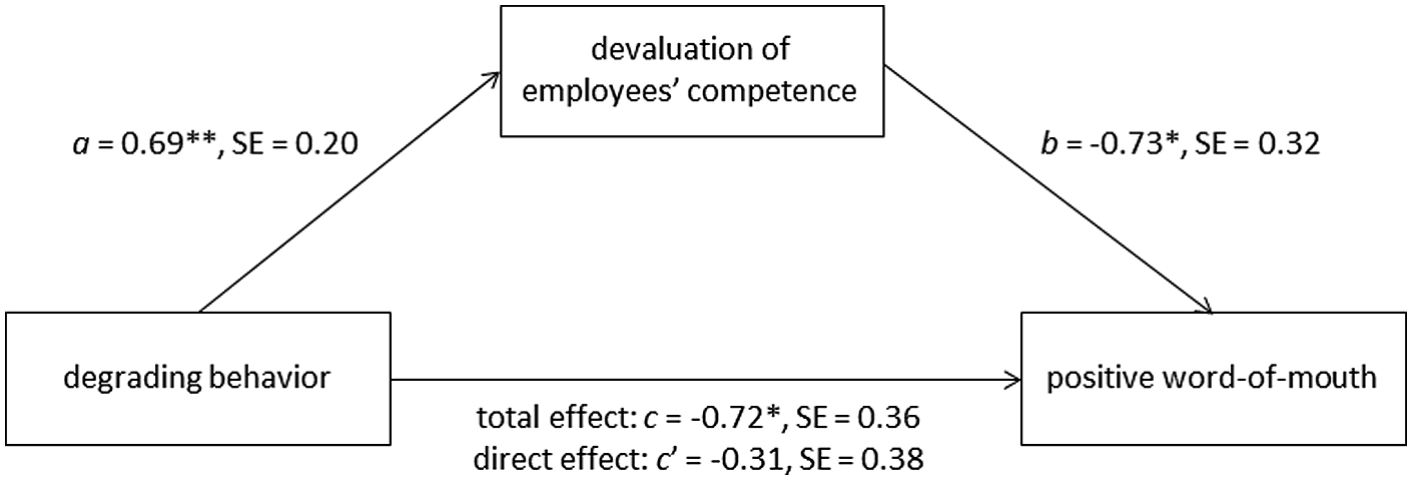
**Devaluation of employees‘ competence mediated the effect of degrading behavior on positive WOM in study 3 (sub-sample complaints).** The mediation analysis was conducted using PROCESS (model 4, Hayes, 2013, p. 445). The mystery callers indicated whether they would recommend the service hotline or not by answering “yes” (coded 1) or “no” (coded 0) in order to measure positive WOM. *a* is an unstandardized regression coefficient; *b*, *c*, and *c′* are unstandardized logistic regression coefficients. *p < 0.05, ***p < 0.001.

Finally, we analyzed whether the effect of degrading behavior (independent variable) on positive WOM (dependent variable) could be explained through the devaluation of employees’ competence (mediator) using PROCESS (model 4, Hayes, 2013, p. 445). The results are displayed in **Figure 6**. The analysis revealed a significant influence of the degrading behavior on positive WOM, *B* = -0.72, *SE* = 0.36, *p* = 0.046, and the mediator “devaluation of employees’ competence,” *B* = 0.69, *SE* = 0.20, *p* < 0.001. Subsequent analyses of the influence of the mediator on positive WOM showed a significant regression weight, *B* = -0.73, *SE* = 0.32, *p* = 0.022, indicating considerable influence of the mediator on positive WOM. When finally examining the influence of the degrading behavior and the mediator devaluation of employees’ competence on positive WOM concurrently, the effect of the degrading behavior was considerably reduced, *B* = -0.31, *SE* = 0.38, *p* = 0.407. The indirect effect of the degrading behavior on positive WOM through the mediator was highly significant as indicated by the 95% CI (-1.72, -0.07) using 1,000 bootstrap estimations.

### Discussion

Results of study 3 confirmed our expectations. The two hypothesized defense responses of the customer were confirmed. The devaluation of employees’ competence (motivated cognition) was higher in case of (aggressive) complaints compared to non-complaints. In addition, following (aggressive) complaints, customers reduced their intention to engage in positive WOM, whereas following non-complaints, customers were more willing to engage in positive WOM. Finally, the mediation analysis provides evidence for the assumed role of the devaluation of employees’ competence as a mediator in predicting positive WOM. More importantly, the results of study 2 were replicated investigating real customer–employee-interaction. More degrading behavior leads to less positive WOM and this effect was mediated by the devaluation of employees’ competence.

## General Discussion

Building on the assumption that an employee, when confronted with a self-esteem threat in form of a complaint, shows defense responses we proposed a ring-model of vicious circles in customer–employee-interaction, in which we differentiate between three defense responses by the employee: motivation, motivated cognition, and motivated behavior. At first, the service employee is confronted with a probable aggressive complaint that elicits a need for cognitive closure as a motivational response. This motivation then leads to a devaluation of the customer and his/her information as a cognitive response followed by the behavioral response of degrading service reaction. The customer confronted with degrading service reaction in turn devaluates the service employees’ competence as a cognitive response followed by reduced repurchase intention and positive WOM as behavioral responses.

At first, the aim of study 1 was to test all hypothesized defense responses of the employee assumed within our ring-model (see **Figure 1**). In this study, participants were asked to put themselves in the role of a service employee and were confronted with either an aggressively phrased complaint (step 1) or a neutrally phrased complaint. Results revealed, as expected, that closed-mindedness (one aspect of the need for cognitive closure) increased (motivation, step 2) after an aggressively phrased complaint leading to a devaluation of the customer and his/her information (motivated cognition, step 3) and in turn to more preferred degrading service reaction (motivated behavior, step 4). Furthermore, study 2 investigated all hypothesized defense responses of the customer assumed within the ring-model. For this purpose we asked our participants to put themselves in the role of a complaining customer and confronted them either with a degrading service reaction or a favorable service reaction. Results revealed, as expected, that a degrading service reaction (step 4) increased the devaluation of employees’ competence (motivated cognition, step 5) leading to less repurchase intention (motivated behavior, step 6) compared to a favorable service reaction. All in all, results of the first two studies revealed evidence for the single links in our ring-model. However, both studies were conducted in the lab using scenarios for which participants were asked to put themselves in the role of an employee or in the role of a customer. To address this issue, data from a mystery call-evaluation study were analyzed (study 3). The mystery callers evaluated the service hotline of a German airline. They brought forward an aggressively phrased complaint or a non-complaint (e.g., flight booking), evaluated employees’ competence, and indicated whether they would engage in positive WOM. As predicted, the results revealed more devaluation of employees’ competence (motivated cognition) in case of complaints then in case of non-complaints. This devaluation then resulted in decreased engagement in positive WOM (motivated behavior). More importantly, the mystery callers also evaluated the degrading behavior of the service employees which made it possible to investigate whether the results of study 2 were replicable testing real customer–employee-interactions. Results demonstrated, as assumed, that more degrading behavior leads to less positive WOM and this effect was mediated by the devaluation of employees’ competence.

### Theoretical Implications

The presented results replicate several findings with regards to complaint management and related outcomes such as WOM behavior and repurchase intentions. Corresponding with prior reports we found that customer complaints indeed evoke negative evaluations of the customer and in turn lead to rather degrading service reactions (Traut-Mattausch and Jonas, 2007). Furthermore, we could confirm that customers are more willing to spread positive WOM and have higher repurchase intentions after they have received competent service (e.g., Johnson et al., 1998; Brady and Cronin, 2001).

Despite the amount of literature concerning antecedents for positive WOM up to this date the main focus of research has been either on employees’ behavior, on customers’ satisfaction, loyalty or trust, or on organizational conditions (for an overview see de Matos and Rossi, 2008). On the one hand, our studies therefore add to the existing knowledge in that they take into consideration and combine the perspectives of both customers and service employees. On the other hand, the presented studies explain existing results by providing new insights in the interaction between customer and service employee. Our ring-model of the interactive process combines customers’ and service employees’ responses and thereby could show that and how both are interrelated.

One might argue that the interdependence of interaction partners is not new. Indeed, examples like the interpersonal circle show that behaviors of interaction partners depend on each other, e.g., critical behavior of one person encourages distrust of the other (e.g., Freedman et al., 1951; Leary, 1957; Strong and Hills, 1986; Wiggins, 2003). The prediction that complaints negatively affect service reactions could also be just another proof that non-cooperative behavior elicits low intention to cooperate in different contexts (Kelley and Stahelski, 1970; Van Lange and Visser, 1999). Or our results could be simply explained by the fact that unpleasant interaction partners evoke matching behavior, i.e., people lower their own level of pleasantness in return (Burgoon et al., 1995) and respond in a hostile and aggressive way (Menon and Dubé, 2000).

However, our analyses went one step further. We could show that the reasons for service employees’ degrading service reactions following complaints are more than only adapting to customers’ rude behavior. The reason is rather an underlying psychological process: Complaints are perceived as unpleasant and threatening events and evoke service employees’ need for cognitive closure. Consequently, the feedback is processed in a closed-minded and biased manner resulting in a devaluation of the customer and his/her information and thus in degrading service reaction. The proposed ring-model of the interactive process of complaint management hence not only describes, but also explains how customers and service employees react upon each other and, eventually, both determine the outcomes of their encounter. Based on that knowledge it should be possible to create effective intervention strategies to improve the customer–employee-interaction.

### Limitations and Future Research

In our research we investigated the ring-model assuming that during the interaction between a complaining customer and a service employee, customer’s behavior (step 1) affects employee’s emerging motivation (step 2) which then leads to employee’s motivated cognition (step 3) and motivated behavior toward A (step 4). Accordingly, employee’s behavior affects customer’s emerging motivated cognition (step 5) and motivated behavior toward the employee (step 6). The motivation “need for cognitive closure” should be responsible for the motivated cognition and for the motivated behavior shown by the employee. However, no motivation is specified for the motivated cognition and the motivated behavior shown by the customer within the ring-model. Results of study 2 demonstrated that degrading service behavior (step 4) elicit a devaluation of employees’ competence (step 5) which could be a cognitive defense response. We therefore suggest that this effect could be explained through *defense motivation* (Chaiken et al., 1989) and thus assume that the effect of degrading service behavior on devaluation of employees’ competence should be mediated by defense motivation. In further research this assumption should be tested and the ring-model—based on empirical evidence—expanded.

We investigated the ring-model of vicious cycles in customer–employee-interaction by building on the Loop2Loop model, which is a dynamic model of social interaction (Jonas, 2015; Jonas and Steindl, 2015; Jonas and Bierhoff, in press). However, not all steps of the Loop2Loop model were transferred to the ring-model such as the motivational-affective state (e.g., physiological arousal) or the specific cognitive focus (e.g., specific goal) of both interaction partners. It would be interesting in future research to also include these additional variables of the Loop2Loop model into the investigation of customer–employee-interactions as well as to further explore the dynamic nature of the resulting social interaction to receive a broader picture of the underlying process of vicious cycles of customer complaints.

As illustrated in **Figure 2** we distinguish four types of behavioral expressions (see **Figure 2**): (A) neutrally phrased non-complaints, (B) neutrally phrased complaints, (C) aggressively phrased complaints, and (D) aggressively phrased non-complaints. In our research we compared the crucial type (C) aggressively phrased complaints to the type (B) neutrally phrased complaints in study 1, as well as to the type (A) neutrally phrased non-complaints in study 3. In future research type (C) aggressively phrased complaints should be compared to type (D) aggressively phrased non-complaints. Based on our argument that a complaint can be seen as negative feedback and therefore threatens the self-esteem of a service employee we propose that service employees respond with an increased need for cognitive closure (motivation), with an increased devaluation of the customer and his/her information (motivated cognition), and with an increased degrading service behavior when confronted with an aggressively phrased complaint compared to an aggressively phrased non-complaint. Moreover, our assumptions would ideally be addressed in future research by comparing all types of behavioral expressions in one study.

A further limitation of our research is the fact that the data in all studies rely on self-report from the same source (except for the second pilot study). Therefore, we cannot rule out that parts of our effects could be driven by participants’ consistent responses across the used self-report measures. In further research it would be important to address this limitation by using psychophysiology measures (threat specific peripheral neurophysiological responses, cf. Blascovich and Mendes, 2010) in combination with real behavior. For this purpose, the psychophysiology response from the service employee as well as from the customer should be measured during an interaction whereas the interaction itself should be videotaped and analyzed afterward by trained raters based on a coding system. This procedure would ensure in our view to investigate customer–employee interaction on genuine interaction date and provide the possibility to test the proposed underlying process—the threatening effect of customer complaints.

### Practical Implications

Positive WOM is spread to five other people whereas in case of dissatisfying events negative WOM warns nine other people, i.e., potential new customers (Knauer, 1992). This fact demonstrates how important it is to foster recommendations. Taking into account that people trust most in personal recommendations (Murray, 1991) and considering the enormous social communication networks online positive WOM even gains in relevance. As our results show, positive WOM decreases in case of complaints due to perceived incompetent service and employees’ degrading behavior toward customers. Successful complaint management therefore needs to focus not only on formal guidelines for service employees or skills training, but also on the fundamental mindset that leads to the defense service reaction. The perception of complaints as visible sign of errors that are to be avoided needs to be replaced by the belief in complaints as valuable chance to satisfy and retain a customer. Thus, trainings should support service employees in reducing the need for cognitive closure and degrading reactions in order to deliver high quality complaint management. In this context Moskowitz et al. (1999) proposed the possibility of inhibiting an automatic motivation in case of complaints by another automatically activated goal. Employees’ need for cognitive closure might thus be inhibited by another equal motivation such as to be curious in order to collect customer ideas within complaints for improvement.

Failures and failing recoveries decrease satisfaction (e.g., Bitner et al., 1990) and are the key drivers for complainants’ switching behavior (Keaveney, 1995). However, successful complaint management offers the chance to restore satisfaction: Successful recovery can even achieve higher satisfaction than prefailure satisfaction levels (for an overview see de Matos et al., 2007) and in the aftermath increase the complainants’ willingness to talk about this positive experience, i.e., spread positive WOM (Swanson and Kelley, 2001).

## Conflict of Interest Statement

The authors declare that the research was conducted in the absence of any commercial or financial relationships that could be construed as a potential conflict of interest.

## Footnotes

1 Psychosocial stress is widely known to induce various adaptive responses of physiologic systems with particular increasing activities in the hypothalamus-pituitary-adrenal axis (HPA) as well as in the sympathetic-adrenal-medullary (SAM) system. Schommer et al. (2003) emphasize that rapid habituation of HPA responses after repeated exposure to stressful stimulation is a frequently reported characteristic of the HPA axis. The authors presented evidence suggesting that with repeated psychosocial stress, a dissociation of HPA and SAM response patterns can be observed, characterized by a different temporal profile of habituation of catecholamine responses as compared to the HPA axis (Schommer et al., 2003). Suzuki et al. (2003) investigated the interaction between coping skills and psychophysiological reactions on stress using cardiovascular responses and SCL level. They reported that effort-distress coping intensified both cardiovascular responses as well as SCL. Therefore, we measured HR and SCL to investigate the stress response of our participants.

2 All laboratory studies (pilot studies 1–2, main studies 1–2, study reported within the discussion part of study 1) were conducted before the ethical board at the University of Salzburg was established in 2011. Nevertheless, treatments of the participants were in accordance with APA ethical standards. All laboratory studies were undertaken with informed consent of the participants. Within the field study 3 we analyzed data from a mystery calls-evaluation survey. This survey was conducted by a professional service provider for mystery calls on behalf of a German airline in order to evaluate the service hotline of the airline. This is a common procedure for research purposes in marketing sciences (e.g., Wilson, 1998, 2002; Finn, 2001; Van der Wiele et al., 2005). Anonymous customers, the mystery callers, pretend to be real customers in order to evaluate the service quality of a service hotline. When they start their position, employees consent to the evaluation of their service behavior in general. At the given moment, however, it was not evident for employees whether the call was “real” or a mystery call.

3 We tested whether the order of presentation of the within-factor has an effect. Results revealed no effect, *F*s < 1.

4 Research conducted by Thórisdóttir and Jost (2011) showed that the manipulation of threat motivated the aspect “closed-mindedness” of the need for cognitive closure. We therefore decided to measure this aspect as well. However, the need for cognitive closure is defined as an “individual’s desire for a firm answer to a question” (Kruglanski, 2004, p. 6). Transferred to a complaint situation a high need for cognitive closure would imply the desire to come to the self-esteem beneficial conclusion that the customer is responsible for the problem which represents the firm answer to the question “who is responsible for the complaint.” Consequently, the service employee should not be motivated to dispute about the complaint (Traut-Mattausch and Jonas, 2007). Thus, we think that the item “how much would you like to talk to the customer (recoded)” reflects closed-mindedness as one aspect of the need for cognitive closure adapted for a customer–employee-interaction.

5 Moreover, we tried to manipulate the identification of the participants within this study. Due to this purpose further variables were measured. However, results of the manipulation check indicate that the manipulation was not successful.

6 Due to the purpose of the evaluation study further variables were measured.

7 It was not possible to record the calls. Therefore, it was not possible to check whether the complaints were more aggressive than the non-complaints.

8 In two cases there were missing data.
